# Adaptive hypofractionted and stereotactic body radiotherapy for lung tumors with real-time MRI guidance

**DOI:** 10.3389/fonc.2023.1061854

**Published:** 2023-01-27

**Authors:** John M. Bryant, Austin J. Sim, Vladimir Feygelman, Kujtim Latifi, Stephen A. Rosenberg

**Affiliations:** ^1^ Department of Radiation Oncology, H. Lee Moffitt Cancer Center & Research Institute, Tampa, FL, United States; ^2^ Department of Radiation Oncology, Comprehensive Cancer Center – The James Cancer Hospital, Columbus, OH, United States

**Keywords:** lung, MRI, radiation, adaptive, radiotherapy, MRI guidance, MR-guided radiation therapy, image-guided RT

## Abstract

The treatment of central and ultracentral lung tumors with radiotherapy remains an ongoing clinical challenge. The risk of Grade 5 toxicity with ablative radiotherapy doses to these high-risk regions is significant as shown in recent prospective studies. Magnetic resonance (MR) image-guided adaptive radiotherapy (MRgART) is a new technology and may allow the delivery of ablative radiotherapy to these high-risk regions safely. MRgART is able to achieve this by utilizing small treatment margins, real-time gating/tracking and on-table plan adaptation to maintain dose to the tumor but limit dose to critical structures. The process of MRgART is complex and has nuances and challenges for the treatment of lung tumors. We outline the critical steps needed for appropriate delivery of MRgART for lung tumors safely and effectively.

## 1 Introduction

**Figure 1:**
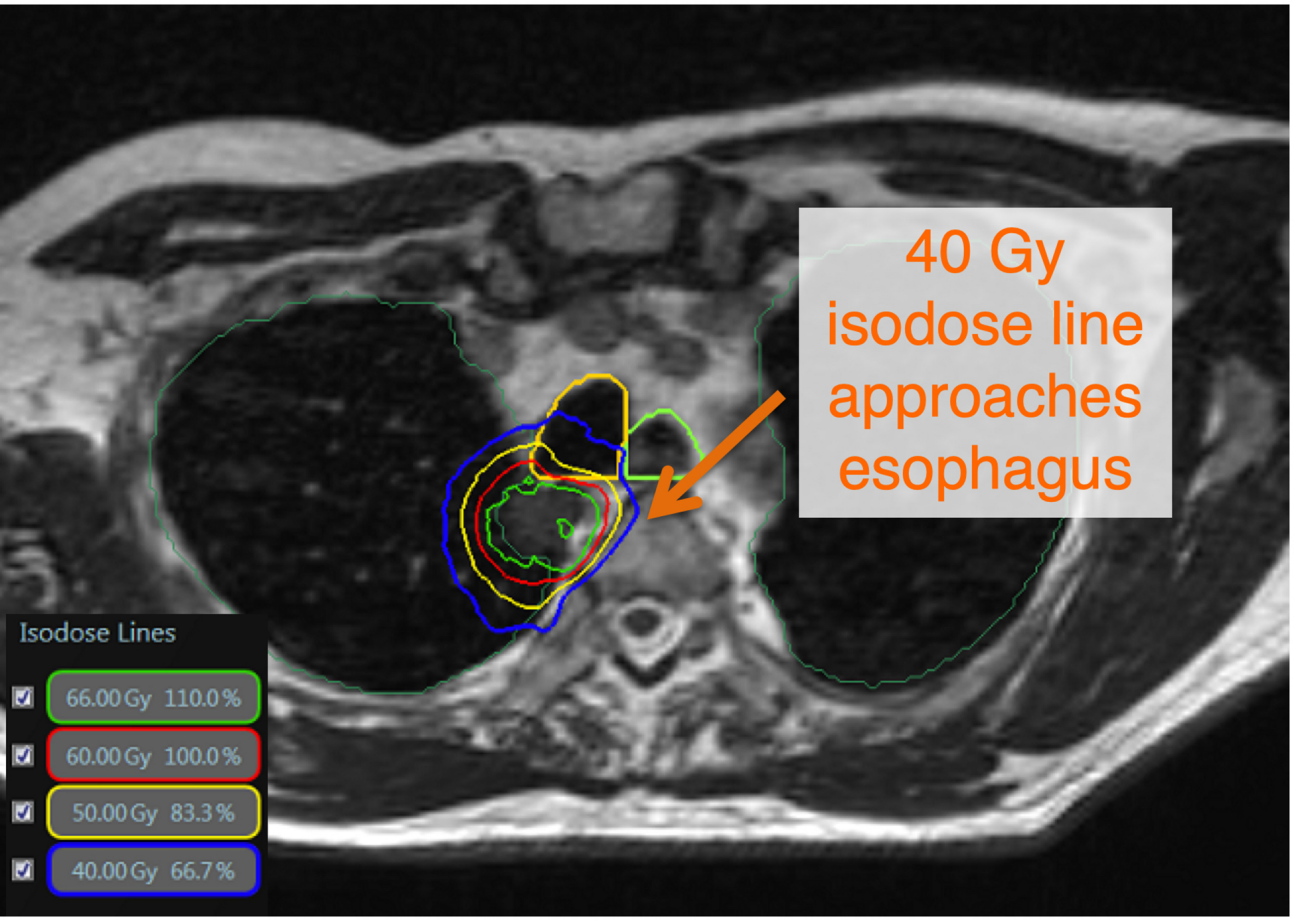
There is no consensus definition of ultracentral lung tumors. The Nordic-HILUS trial defines ultracentral targets as ≤ 1cm from the proximal bronchial tree, which is also used at our institution. These close tumors may lead to high dose to critical organs such as proximal airways and the esophagus. This is a TRUFI sequence on the MRIdian system showing an ultracentral tumor with doses approaching critical organs such as the esophagus and trachea (11).

Despite the widespread use of stereotactic body radiation therapy (SBRT) and image guided radiotherapy (IGRT) for the treatment of primary and metastatic lung tumors (1–6), central (7, 8) and ultra-central lesions (9, 10) remain a therapeutic challenge for safe delivery of ablative radiation doses using conventional linear accelerators. Specifically, ablative doses near central structures, such as the esophagus, proximal bronchial tree, and great vessels have generated concerning toxicity signals in prior randomized controlled trials, even with slightly lower doses per fraction (9) (**Figure 1**). Indeed, in the recently reported Nordic-HILUS trial, delivery of 56 Gy in eight fractions to lesions adjacent to these central structures resulted in a grade 5 toxicity rate of 15.4% (9). The toxicity in the Nordic-HILUS trial may be secondary to significant hot spots (150% of prescription), heterogeneity in organs at risk (OAR) segmentation, and not delineating the walls of critical organs (i.e. mainstem bronchi) (12). Although there is no consensus for defining ultracentral lung tumors, our institution has adapted the Nordic-HILUS trial’s definition of ≤ 1cm from the proximal bronchial tree. The highest risk patients are those with tumors within 1 cm of the trachea and mainstem bronchi (Group A in the Nordic-HILUS trial) as they had the highest risk of death from treatment.

**Figure 2:**
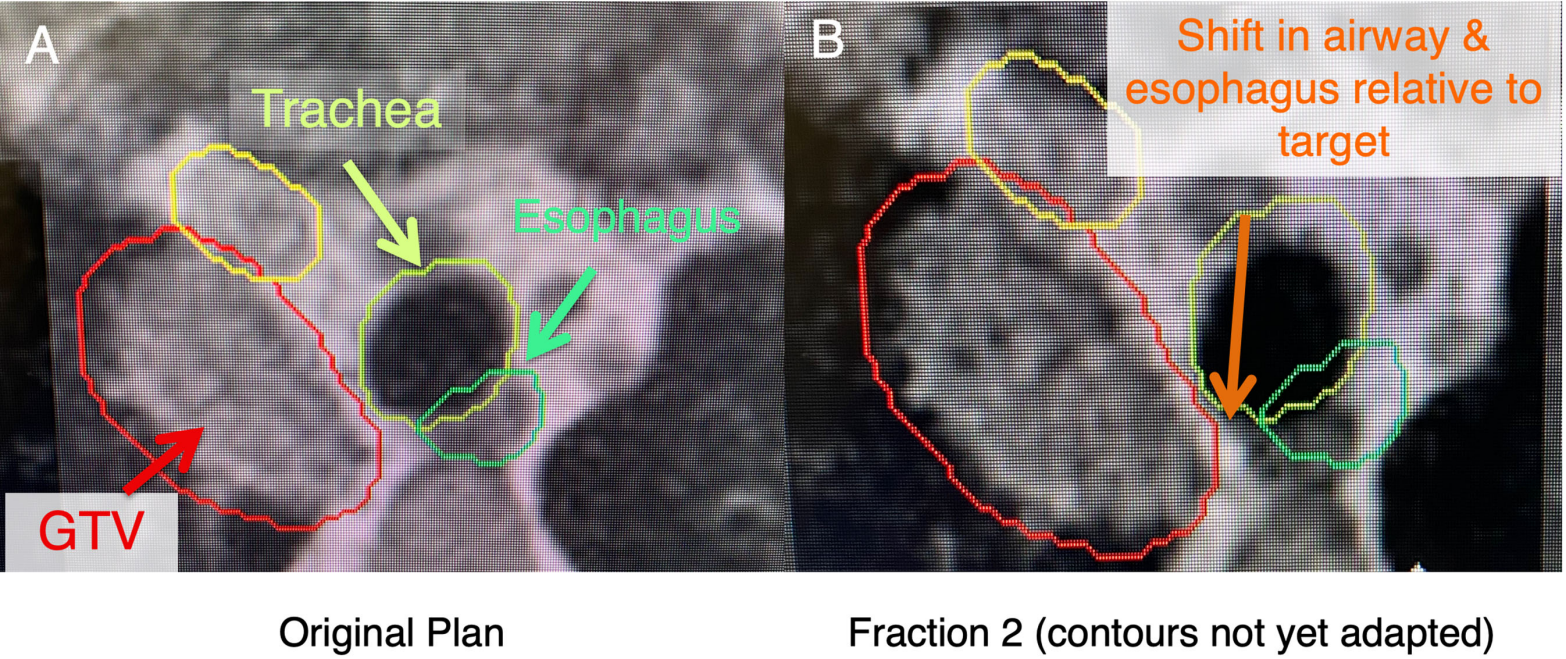
In these ultracentral locations, there may a shift of anatomy secondary to setup uncertainty and movement of nearby critical organs (i.e., esophagus), which may lead to an unacceptably high dose to these critical organs. **(A)** shows a patient with an ultracentral lung cancer at MR-simulation. **(B)** shows a shift of the esophagus and trachea relative to the tumor before contours are adapted (changed to the anatomy of the day).

Respiratory and cardiac motion during radiotherapy necessitates adequate motion management strategies to account for tumor movement (13–15). An example includes the use of an internal target volume (ITV) approach that results in larger treatment volumes (16). These larger volumes may increase overlap with critical OAR which may increase the rate and severity of potential toxicities. This necessitates a trade-off between toxicity and potential for local control in high-risk locations (17). These are critical considerations in the central/ultracentral locations due to the movement of OAR or slight changes in set up that alter the airway position in relation to the tumor (**Figure 2**). With standard IGRT, imaging and beam delivery are typically not simultaneous and must be delicately balanced for the optimization of dose placement, which represents a significant daily challenge for many clinicians (18). However, the advent of magnetic resonance (MR) image-guided adaptive radiotherapy (MRgART) has demonstrated promise in mitigating many of these impediments, leading to the best of both worlds: ensuring adequate ablative dose, while at the same time minimizing OAR doses to unprecedented levels (19, 20).

In this paper, we will review the workflow that our institution uses to perform MRgART for central and ultracentral lung tumors. This workflow incorporates the MRIdian (ViewRay Technologies Inc, Oakwood Village, Ohio), a 0.35T MR linear accelerator (MRL), that has a unique real-time tracking feature. This is important because the motion management enabled by this feature underpins this process.

## 2 Patient selection

Radiotherapy always requires careful patient selection, however, due to the functional design and geometry of MRgRT systems, proper patient selection requires additional considerations prior to simulation. The key aspects include (1) body habitus and (2) presence of claustrophobia, given the narrow bore (70 cm diameter) of both commercially available MRL systems (21, 22). While there are no current effective strategies to deal with body habitus, many patients can tolerate MRgRT with low-dose anxiolytics. The (3) presence of MR-incompatible devices and implants must also be considered and every institution utilizing MRgRT should implement an effective MRI safety screening protocol. Patients must also be able to (4) lie flat and (5) hold their breath for at least 25 seconds for the simulation scan, in addition to longer time intervals for treatment delivery, particularly when respiratory gating is used for motion management. Treatment times are significantly longer when using an adaptive workflow, and therefore, patients must also (6) be able to extend their arm(s) cranially for extended periods of time, which can be more than 60 to 90 minutes.

## 3 Simulation

When scheduling for simulation (SIM), care must be taken to schedule both an MR simulation for target delineation, as well as a computed tomography (CT) simulation to acquire electron density data. These scans are preferably performed in immediate succession during the same patient visit to obtain the most accurate image registration for subsequent dose calculations. Patients are taken to the MRL and placed in a supine position between the flexible body coils, with their arms up on an MR-safe wing board. Although having both arms cranially extended is ideal to ensure the maximum number of possible beam angles, patients with limited mobility and/or other range of motion limitations may be simulated with one arm up (ipsilateral to the tumor), or in the worst case, with both arms by their side. No further immobilization is typically required. During simulation, a 25-second 3D balanced fast imaging with steady-state free precession (TRUFI) sequence (23) is obtained while the patient performs a deep inspiratory breath hold (DIBH). A representative sagittal slice containing the primary tumor is identified. This region of interest (ROI) is then contoured on three consecutive sagittal slices to create a tracking structure. A 3 mm isotropic expansion of the tracking structure is then created to form a “boundary structure” (i.e., gating envelope) for real time gating during treatment delivery at our institution. The gating boundary structure should be completely encompassed by the planning target volume (PTV) to ensure appropriate dosimetric coverage. A 25-30-second cine sequence is obtained while the patient is performing cycling of breath hold and free breathing maneuvers to ensure appropriate tracking and duty cycle for treatment delivery. A percentage excursion threshold of the tracking structure (i.e., primary tumor) outside of the boundary structure is typically set at <5% to trigger beam on at our center. Other institutions utilize a <5-20% trigger for beam on as long as the PTV margin is bigger than the boundary structure—for example if the gating structure is the ROI (most commonly the tumor) expanded by 3 mm and the PTV margin is say 5 mm (20). The patient is subsequently marked at the laser sites and taken to the CT simulator after MR sim.

The patient is then placed in an identical supine position as they were the MR-Linac at the CT simulator, complete with dummy coils, and undergoes a deep inspiratory breath hold scan (DIBH). It should be noted that this contrasts with a 4D CT scan that is typically done for conventional SBRT when using an ITV approach. Some centers utilize a shallow breath hold technique or a mid-respiratory cycle approach. Our center uses DIBH if it is tolerated by the patient. If this is not tolerated, we will often use a free breathing approach. There has also been increased interest across centers to utilize 2-3 L of nasal oxygen during treatment to improve tolerability and gating duty cycle for treatment. We currently use this intermittently as needed.

## 4 Contouring

A static breath hold MRI is used to contour the gross tumor volume (GTV) because MR guidance can utilize real-time gating. Although the MR is the primary data set for contouring, the CT can be valuable to help delineate tumor spiculations and OAR (e.g., airways). Therefore, the appropriate fusion of the CT and MR images is critical for appropriate tumor and OAR delineation. The GTV is isotropically expanded by 3 mm to create the nominal planning target volume (PTV) and is equivalent to our boundary structure.

**Figure 3:**
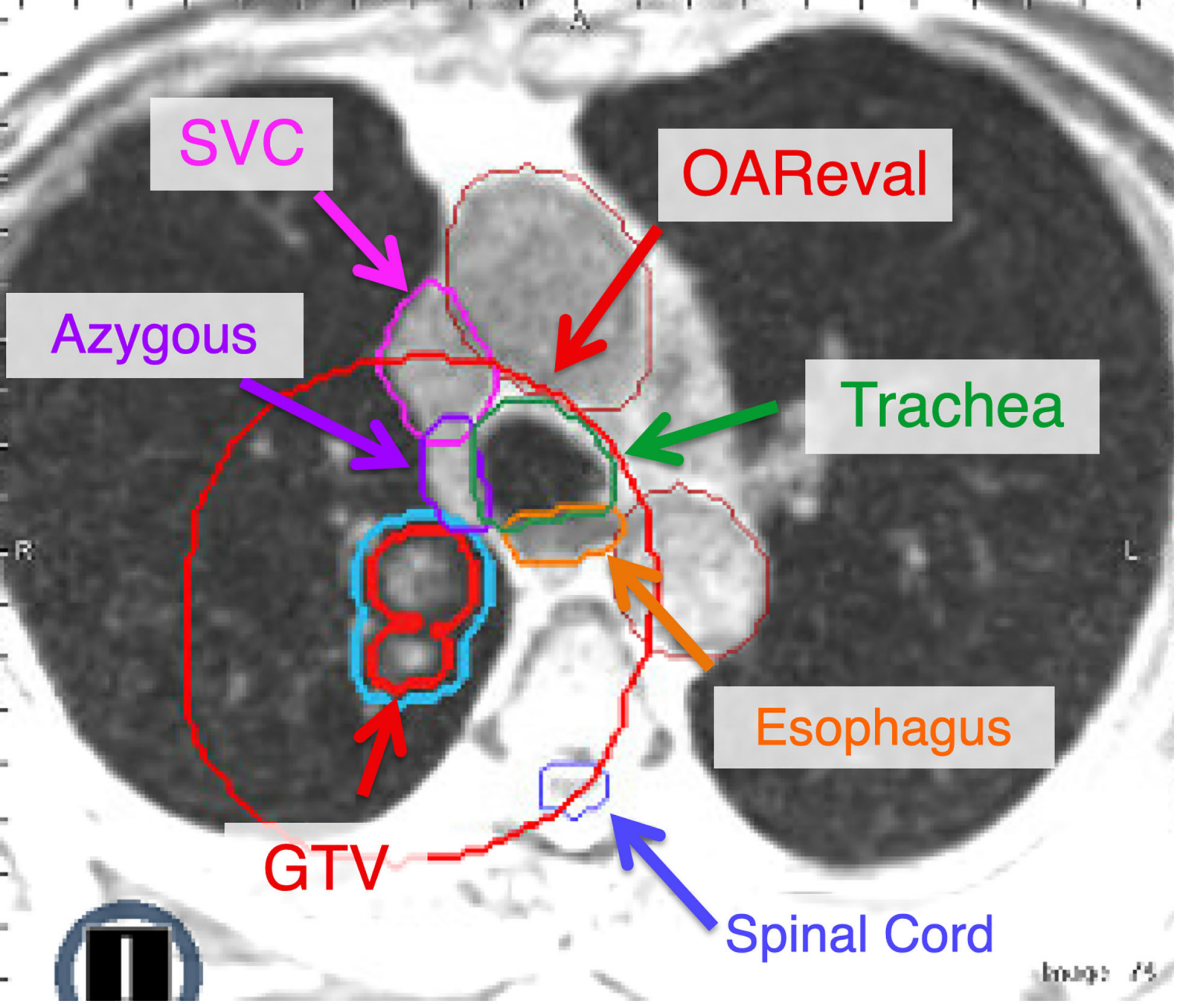
The OAReval structure is generated by taking the PTV and expanding 2 cm sup/inf and 3 cm radially. Central and ultracentral structures should be contoured to ensure inclusion of the wall of critical structures such as the esophagus and airways (i.e., trachea, mainstem). The focus of adaptive recontouring will be on OAR within the OAReval structure. However, it is critical to ensure an appropriate spinal cord contour is present. At our institution, we recommend contouring all great vessels (Aorta, SVC, etc.). However, we also include the brachiocephalic and azygous if they fall within the OAReval to ensure that they do not receive doses above 105% of the prescription.

A key aspect of contouring these structures is appropriate contouring of OAR. Most importantly, this means including the walls for tubular critical structures such as the esophagus and proximal bronchial tree. The contour of these OAR should not be only the air within these structures, as this may lead to incidental hot spots within the walls during initial planning or adaptation. The proximal bronchial tree includes the distal 1/3 of the trachea, mainstem bronchi and lobar bronchi until segmental bifurcation occurs. For simplicity, we often include the entire trachea as part of the proximal bronchial tree contour. The entire course of the esophagus should be contoured through the thorax. Again, it is critical that the wall of the esophagus is included in this contour. The great vessels are often underappreciated in contouring these cases. It is important to include the walls of these vessels to ensure hot spots are not being placed there. Although we include great vessels as part of our contours (aorta, superior vena cava and pulmonary artery), we will also contour out the brachiocephalic vessels and the azygous vein if within 2-3 cm of the target (**Figure 3**).

The PTV is then expanded by 2 cm sup/inf and 3 cm radially to create an “OAR eval” structure, within which the OAR will be recontoured daily for adaptive treatment. OAR that require contours within this ring are any critical structures that include the lungs, spinal cord, chest wall, heart, esophagus, proximal bronchial tree (PBT) (7), and the brachial plexus if indicated, as they are in conventional lung SBRT plans. Additionally, the great vessels (i.e., aorta, superior vena cava, and pulmonary artery) are typically contoured separately to extend at least 2 cm beyond both the cranial and caudal extent and, in addition, 3 cm beyond the radial extent of the PTV (**Figure 3**). At our institution, we are not typically recontouring the GTV daily for these patients. However, if there is a change in tumor volume secondary to necrosis/edema, this may necessitate adjusting the GTV contour and recreating the PTV (with the same 3 mm isotropic expansion). Of note, there are some centers that recontour the GTV daily with each fraction.

**Figure 4:**
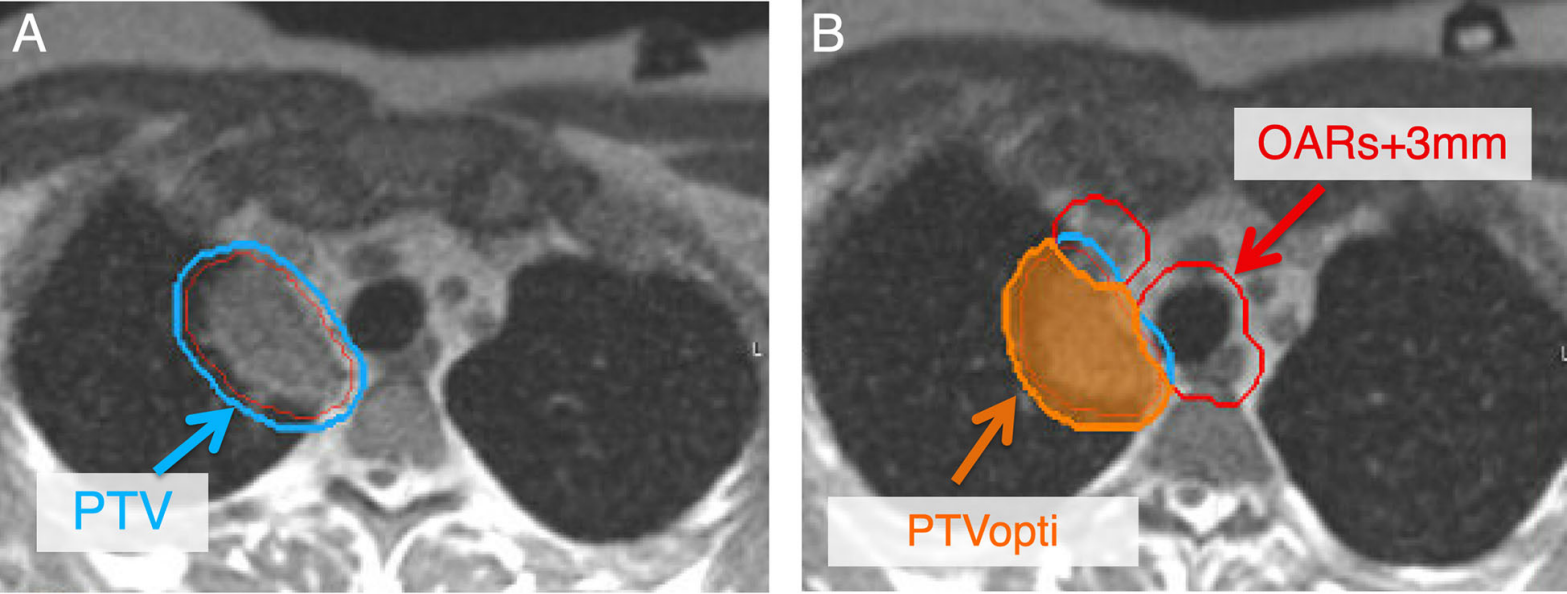
Secondary to changes in position to critical OAR, adaptive planning is pursued to decrease doses to critical OAR while maintaining high dose to the tumor. To develop an adaptive plan, a PTVopti (PTVoptimization) is generated for the optimizer to re-plan based on the anatomy of the day. The PTV at our institution is a 3 mm expansion from the GTV **(A)**. The PTVopti is generated by taking the critical OAR and expanding them by 3 mm—we then take the PTV minus the OAR+3mm to generate the PTVopti **(B)**. This allows for appropriate fall off dose toward critical OAR.

Central OAR that may trigger adaptation, including the PBT, great vessels, and esophagus are combined into a single structure and expanded by 3 mm to create an avoidance structure (OAR+3mm). This avoidance structure is then subtracted from the nominal PTV to generate a PTVopti structure that drives the optimizer and can be modified by the daily adaptation process. The Boolean logic on the structures that are expected to change daily is saved as rules that can be easily applied during adaptation. Since all structures must have placeholders prior to daily adaptations, the appropriate density control structures are always added. At our institution, we use densWater, densAir, and densOther structures as needed (**Figure 4**).

## 5 Planning

Our institutional practice is typically to treat the central and ultracentral lesions to either 50 Gy in 5-10 fractions, 60 Gy in 8 fractions, or 60 Gy in 15 fractions, depending on the histology and anatomic location. The most common dose and fractionation is 60 Gy in 8 fractions. Our goals are to ensure we approach a biological effective dose (BED) of at least 100 Gy for these regions while respecting OAR tolerances (24). We limit the Dmax (single voxel, 2 mm isotropically) within these tumors to 120-125% of the prescription dose. For critical OAR such as the great vessels and the PBT when delivering 60 Gy in 8 fractions, we try to limit the Dmax to 105% of the prescription. To be conservative, we try to limit the esophagus to 40 Gy Dmax over 8 fractions (**Figure 1**). Previous studies have shown that D1cc<40 Gy to the esophagus has a low risk of toxicity with 8 fraction treatment (11). The importance of low esophageal dose in central and ultracentral tumors is critical as esophagus may be associated with significant motion between fractions (**Figure 2**). These dose constraints are consistent (and may be more conservative in some instances) than those used in the SUNSET trial (NCT03306680). These plans typically have approximately 15-18 beams and 50 segments although there can be significant variance depending on lesion size, OAR locations, and if stricter OAR constraints are utilized by the physician due to increased concern of toxicity.

In addition to standard dose constraints for OAR dependent on the dose/fractionation and the anatomy of the day, additional pre-defined metrics are used as thresholds to trigger daily adaptation. While every effort is taken to obtain the optimal plan, the option of daily online adaptation allows for some additional flexibility in accepting suboptimal plans *a priori* with the understanding that online adaptation allows for incremental optimization based on the anatomy of the day. The goal of treatment is isotoxic dose delivery to tumor, i.e., ensuring a maximum cumulative BED during a course of treatment while minimizing toxicity.

It is a good strategy to have a standardized plan labeling strategy for the final approved base plan and each adaptive plan to minimize confusion. For example, there could be up to nine total plans for a patient if they are being treated with 60 Gy in 8 fractions. At our institution, the final approved base plan is appended with the suffix notation of “_A0.” Adaptive plans will thus be labeled in iterative succession with the trailing integers representing the fraction number. It is not necessary to use this labeling system but utilizing a standardized system that is understood by all users will help minimize errors in proper plan identification.

## 6 Technical considerations

Although MRgART has many advantages, including tighter margins, real-time gating, and adaptive replanning, significant challenges remain to overcome central and ultracentral lung lesion treatment limitations. This includes the low proton density of the lung and artifacts secondary to air-tissue interfaces in addition to both cardiac and respiratory motion (25).

At this point, treatment planning for lung malignancies requires a CT scan in the same respiratory phase as the planning MRI (see above for simulation). MRI-only planning with a “synthetic CT” is hard to implement in lung where the density can vary significantly (i.e., between 0.02 to 0.3 g/cm^3^) depending on the patient characteristics and respiratory state (26). While MRI-based segmentation could differentiate between air, lung, tissue and bone, a CT is needed to estimate the lung density, which in turn affects the tumor coverage and the normal lung dose (27). The bulk lung density assignment is definitely not dosimetrically appropriate and it remains to be seen if machine learning approaches could eventually become reliable in determining the actual lung density (28). As of now, the thorax is not a region that is believed to be feasible initially with MRI-only planning techniques (29).

Deformable image registration in MRIdian is usually sufficiently accurate to properly align the soft tissue/tumor, lung, and airways between the MRI and CT datasets. To achieve a more accurate deformable registration, the MR and CT scans should be done in the same setup and in a relatively short time from each other, preferably with MR sim being done first with CT reproducing its setup shortly after. Unlike in the abdomen, manual replacement of air density with tissue or vv. is virtually never required. The standard motion management strategy is a combination of breath hold with real time tumor gating technique with either fixed gantry angle 3D or step-and-shoot intensity-modulated RT (IMRT) beam delivery. The intrafractional MR cine of the MRIdian provides sufficient real time tumor motion visibility to accurately gate the tumor directly without relying on any indirect techniques despite magnetic interference of nearby electrical motors. This method results in the least amount of normal tissue irradiation compared to other motion-management techniques, but it is also the slowest (30, 31).

## 7 Daily adaptive workflow

At our institution, the steps of the MRgART workflow outlined below are embedded in formal checklists that are followed during both the initial planning and adaptive treatment phases. We encourage any new center looking to develop and implement an MRgART program to develop similar checklists to ensure each step in the adaptive processes is followed in a consistent manner.

### 7.1 Positioning

Daily online adaptation is performed while the patient is positioned on the table reproducing the simulation set up. A new 3D MR scan of the day is obtained with the field of view including the entirety of the target with a superior/inferior margin and the entirety of corresponding patient anatomy. Positional adjustments are limited to 3D translations only. The translational shifts are based on manual primary tumor alignment and approved by the physician. An external contour corresponding to the new patient scan/position is automatically generated. It serves as a guide for the system to determine if the shifts can be executed safely. Once confirmed, the shifts are executed. The target volumes are always rigidly translated and aligned from the simulation MR to the daily volumetric MR scan frame of reference. The OAR also need to be segmented on the daily MR. At our institution, we found that rigid registration has been the most consistent starting point in the thorax as compared to deformable registration. The electron density map from a simulation CT deformably registered to the daily MR is examined and necessary overrides, if any, could be performed using the pre-defined density control structures.

### 7.2 Adaptive re-contouring

The tracking structure, which is usually based on the GTV, is then evaluated, and modified if necessary by a properly trained radiation therapist. To save time, the OAR are typically edited only within the bounds of the focused OAReval ring. However, the spinal cord is always segmented, and dose verified even if it lies outside the OAReval structure. Although the rigidly translated lung contours do not need to be perfect on the daily MR, they should reasonably approximate the daily anatomy to ensure that lung dose is appropriately accounted for. At our institution, adaptive OAR contouring is a team effort, initially completed by the radiation therapist or radiation oncologist trainee, followed by a thorough review by an attending physician with experience in adaptive radiotherapy.

GTV volume edits are usually not required because plan contours are aligned to the target on the daily MR image and there are typically minimal geometric changes of the target over the course of treatment to justify edits to the GTV (i.e., the original GTV is able to encompass the target). However, GTV edits are necessary if target geometry has changed enough where the original contour no longer appropriately delineates the tumor edges or if an interface with an abutting OAR evolves over the course of treatment as such so that the GTV now overlaps with the OAR. We realize there are institutions that do edit the GTV daily for adaptive treatment. In our practice, we have found that with SBRT (8 fractions or less) there is minimal change in the tumor volume over a treatment course that necessitates daily GTV edits (exceptions do exist). If edits are made to the GTV then the PTV volumes must be regenerated as stated above. After the attending physician is satisfied with the segmentation effort, the OAR contours are cleaned up according to the pre-determined software protocol (these settings are user defined and we use them to remove holes, smooth out edges, and remove disconnected contours). Pre-set Boolean rules are then applied to generate the new PTVopti. The nominal PTV remains unchanged during this process, unless the GTV was modified. A useful check of the adaptive process is to watch the PTVopti change with application of the rules.

### 7.3 Dose Prediction

A prior plan, which can be the base plan or a previous adapted plan, is recalculated on the daily imaging dataset taking the isocenter shift into account. The target and recontoured OAR metrics achieved by either the base plan or a prior adapted plan on the daily MR anatomy scan are then evaluated. If any target coverage or OAR constraint violations occur, a decision is made to either pursue a simple weight optimization (i.e., changing the relative distribution of monitor units [MUs] between the beamlets without changing their shape or number) or to immediately proceed to a full re-optimization, whereby the beamlets will change based upon whether the cost function is modified. The beam angles never change. After reoptimization, the new plan may still require manual reoptimization to meet any of the critical metrics, including target coverage or OAR constraint(s).

**Figure 5:**
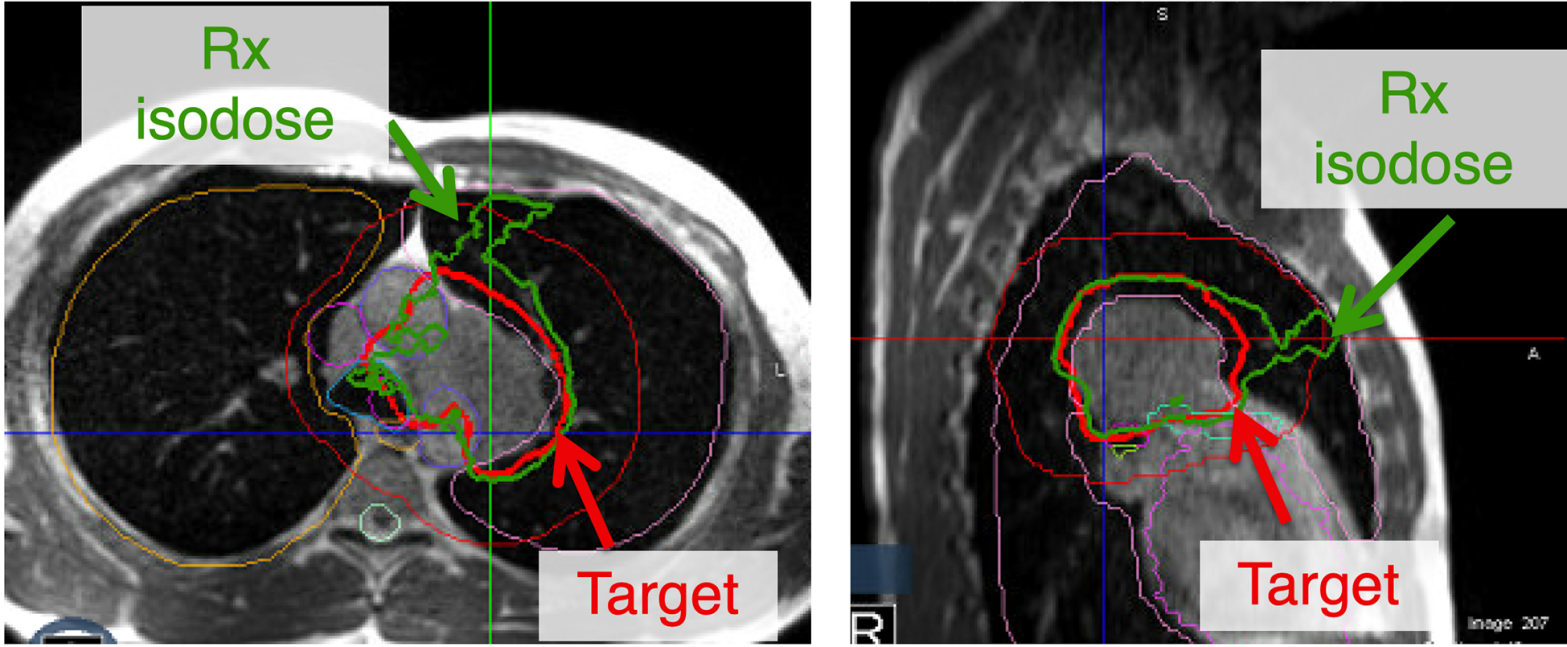
Although all the constraints for adaptive replanning may be met, a critical last step is to evaluate the isodose lines. This may be secondary to contouring errors that could be overlapping into an inappropriate location. Additionally, a lack of conformality to the plan may lead to the prescription isodose line not following the target. In this figure, all the constraints are met but the plan lacks appropriate conformality and another iteration of adaptive re-planning may be warranted.

The original plan generated on the always remains available for treatment should it be chosen after reoptimization. Once an optimal plan is chosen based on the DVH metrics snapshot, the crucial last step is to review the isodose lines through the target level and within 2 cm superiorly and inferiorly. This is a good practice since even if the pre-defined DVH metrics are all met, unexpected hot spots away from the target or lack of coverage conformality could be easily visualized and further corrected by replanning if necessary (**Figure 5**).

At the end of the planning process patient-specific dosimetric quality assurance must be performed as with any inversely planned treatment. It must rely on independent dose recalculation since a pre-treatment measurement is obviously not possible with a patient on the table. To that end, the MRIdian system has a second Monte Carlo calculation engine that relies on a code completely different from the main one. The two dose distributions are compared by gamma-analysis (32). The gamma analysis is performed with 2% (local normalization) dose-error threshold, 2 mm distance-to-agreement threshold, and 10% of the maximum dose analysis cut off threshold. While 95% passing rate with 3%/2mm dose-error/distance to agreement threshold should be considered acceptable (33), in our experience 100% agreement with more stringent 2%/2mm criteria is typically achieved. Also, the total MUs for the daily online adapted plan are compared to the original plan and recorded prior to treatment delivery.

### 7.4 Dose delivery

A MR-compatible monitor is installed on the far wall of the vault where the bore axis intersects it, as a visual aid for coaching the patient to keep their breath held at the needed respiratory position. The monitor replicates the pertinent portions of the operator console screen, most importantly the moving tracking structure and the stationary gating envelope. The patient can see the monitor in a mirror thus receiving feedback on their efforts to hold the breath in the optimal position as instructed by the therapists.

If the anatomy at simulation was not representative (e.g., underinflated lungs) or an optimal plan was not achievable, causing the new online daily adapted plan to be clearly superior, it may be saved as the new default base plan for future fractions. If keeping the tracking structure within the boundary structure proves challenging, the team may elect to liberalize the voxel excursion percentage to above 5% to achieve a practical duty cycle depending on PTV margins and the clinical context.

### 7.5 After the treatment course

**Figure 6:**
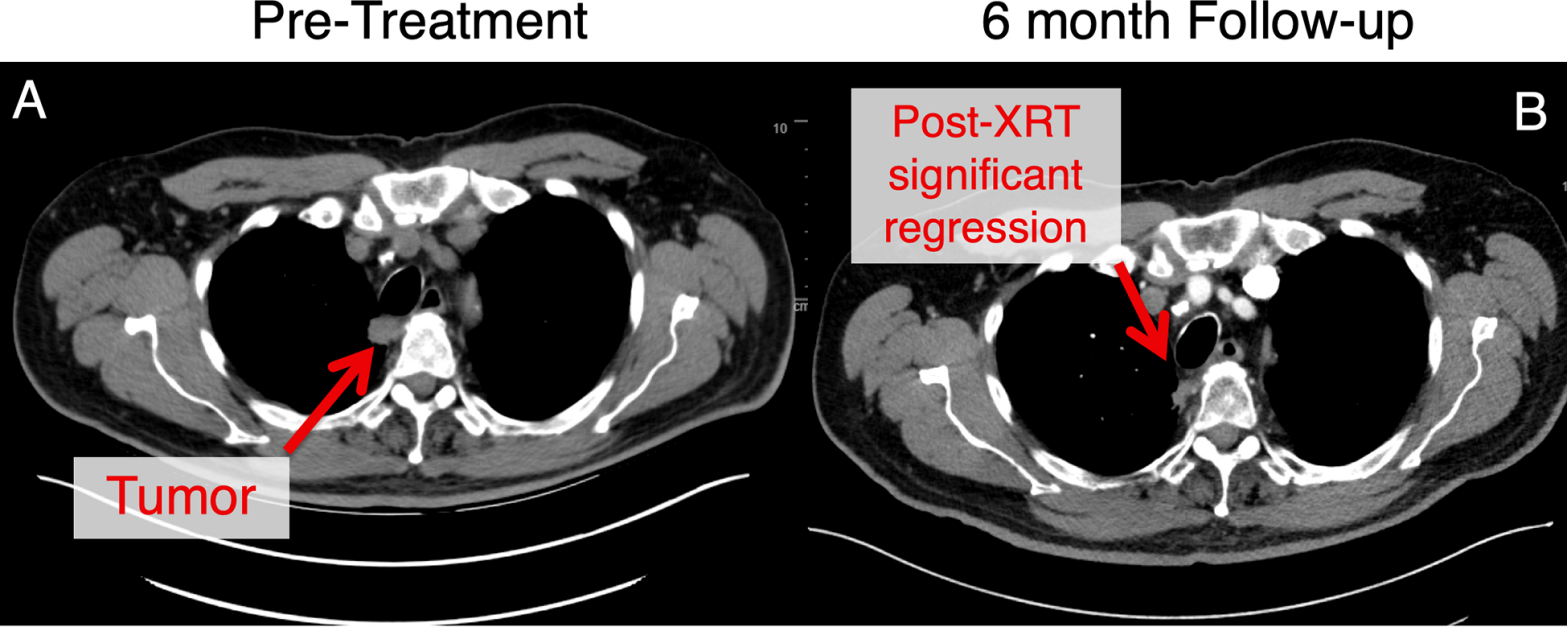
This patient with an ultracentral tumor was treated with 60 Gy in 8 fractions with daily adaptive therapy. This tumor was adjacent to the trachea and esophagus at the start of treatment **(A)**. Follow-up at approximately 6 months **(B)**, there has been significant tumor regression for this patient. This patient experienced mild gastroesophageal reflux disease at baseline that was managed with proton pump inhibitor therapy without any other toxicities noted.

Patients must be closely monitored for toxicity. Per our institutional experience, the approach has been associated with excellent primary tumor control and minimal toxicity with presentations and manuscripts pending (**Figure 6**). However, these patients remain at risk for regional (i.e., lymph node) failure and should be followed closely with serial CT scans.

## 8 Conclusion

MRgART allows for ablative doses to be delivered safely to central and ultracentral lung lesions, to achieve improved local control while minimizing toxicity. However, the proper use of this technique is required to ensure that OAR remain protected from ablative doses. In this guide, we have reviewed our institution’s MRgART workflow that allows us to achieve the necessary target coverage while respecting the OAR’ tolerances. This appears to be a practical and consistent interim approach with MRgART, as we await the results of the prospective LUNG STAAR trial (NCT04917224).

## Data availability statement

The original contributions presented in the study are included in the article/supplementary material. Further inquiries can be directed to the corresponding author.

## Author contributions

All authors confirm contribution to the paper as follows: conception, design and drafting of the manuscript. All authors contributed to the article and approved the submitted version.
